# Framework conditions facilitating paediatric clinical research

**DOI:** 10.1186/1824-7288-37-12

**Published:** 2011-02-23

**Authors:** Anne-Laure Knellwolf, Stéphane Bauzon, Ornella Della Casa Alberighi, Irja Lutsar, Ernö Bácsy, Deborah Alfarez, Pietro Panei

**Affiliations:** 1 Dept. of Therapeutic Research and Medicines Evaluation, Istituto Superiore di Sanità, Rome, Italy; 2 Storia e Teoria del Diritto, Facoltà di Giurisprudenza, University of Rome "Tor Vergata", Rome, Italy; 3 Clinical Pharmacology Unit, G. Gaslini Institute, Genoa, Italy; 4 Dept. of Microbiolgoy, University of Tartu, Tartu, Estonia; 5 Medical Research Council of Hungary, Budapest, Hungary; 6 ERA-NET Priority Medicines for Children Coordination, The Netherlands Organization for Health Research and Development, The Hague, The Netherlands

## Abstract

The use of unlicensed and "off-label" medicines in children is widespread. Between 50-80% of the medicines currently administered to children have neither been tested nor authorized for their use in the paediatric population which represents approximately 25% of the whole European population. On 26 January 2007, entered into force the European Regulation of Paediatric Medicines. It aims at the quality of research into medicines for children but without subjecting the paediatric population to unnecessary clinical trial. This article addresses ethical and legal issues arising from the regulation and makes recommendations for the framework conditions facilitating the development of clinical research with children.

## Introduction

The Coordination of Research on Priority Medicines for Children (ERA-NET PRIOMEDCHILD) is a network of research funding organisations from eleven different European Union (EU)-member-states, initially founded on January 1^st^, 2007 for a period of three years extended to four due to an official extension by the European Community (EC). The network's primary goal is to contribute to the European Research Area on priority medicines for children by implementing a European joint research programme including development and innovation themes [1]. ERA-NET PRIOMEDCHILD's concern is also to bring coherence and cooperation to national research programmes and to establish policies on priority medicines for children research.

## Setting

Between 2007 and 2010, key national and international organisations including the European Medicine Agency (EMA) and its Paediatric Committee (PDCO) representative members have been consulted to address research, research funding, public-private research cooperation, ethical and regulatory issues. A set of workshops and meetings involving stakeholders, i.e., academic experts from public hospitals, pharmaceutical industry's, insurance company's, parent associations' and health authorities representatives were held. Based on expert consultations, questionnaires sent to stakeholders and a literature review on that topic, ERA-NET PRIOMEDCHILD identified legal and ethical issues arising from the European Regulation of Paediatric Medicines and made recommendations for facilitating clinical research with children.

## Results

### Obligations/Derogations for the pharmaceutical industry and their consequences

The three legal pillars of the new Regulation [2] are i) the adoption of incentives for industry; ii) the implementation of a mandatory Paediatric Investigation Plan (PIP) considering all age ranges and iii) the creation of a Paediatric Committee (PDCO). The Regulation provides therefore strong obligations for the pharmaceutical industry together with some rewards and incentives in order to facilitate the development and accessibility of medicinal products in paediatrics. Rules are different for *patent protected *and *off-patent medicinal products*.

For new indications, new routes of administration or new formulations of already *patented products *and for the development of *new medicinal products*, pharmaceutical companies (applicants) have to submit a PIP to the PDCO before Marketing Authorization Application (MAA) is submitted. The PIP binds applicants, but it may be amended. Indeed, for products devoid of interest in children, "waivers" of class or indications or specific products can be requested [3] as well as "deferrals" of initiation of paediatrics studies and/or completion for products for which adult data are needed can be requested for all subsets of the paediatric population. In both cases they must be fully justified by the applicant. For studies conducted in compliance with an agreed PIP and if information is correctly provided, a reward of 6-month extension of the Supplementary Protection Certificate (SPC) will be granted. Besides, the SPC extension duration may slow down the promotion of generic formulations, thereby adding to the health costs [4].

A new type of marketing authorization - Paediatric Use Marketing Authorisation (PUMA) - has been established by the Regulation to stimulate the development of *off-patent products *for paediatric use. If paediatric indications and formulations - based on studies conducted in line with a previously agreed PIP - are authorized, it is possible for the applicant to get a PUMA approval with 10-year market exclusivity. Moreover, community funding for studies on *off-patent products *under the Paediatric Research Programme will be possible. Conversely, submitting a PIP and producing data about the paediatric population for *off-patent products *is an optional procedure.

Not many PUMAs are so far being applied for *off-patent products *which represent an important part of available medicines for the paediatricians [3]. To reduce the prevalence of unmet need for children research, ERA-NET PRIOMEDCHILD will recommend that PUMA procedure should be mandatory and/or other incentives implemented in order to stimulate the paediatric registration of *off-patent products *for children. The regulation should also have included obligations for applicants regarding *off-label drugs *(i.e., drugs administered for a different purpose from that studied and reviewed for licensing) which are very commonly used in ordinary practice. Actually, clinical research on these products is rather complicated and the profit margin being low, pharmaceutical companies generally do not make *off-label drugs *available for children research [5].

### Role of the Paediatric Committee of the EMA

The paediatric committee (PDCO) includes representatives from all EU-member-states, health-care professionals, members of patients' associations, permanent and external experts, methodologists and ethics specialists. Evaluation by the PDCO should take into consideration the significant therapeutic benefits of the proposed studies for children, the need to avoid unnecessary studies and delay of authorization for adult populations. The drug safety and the monitoring of the clinical trials involving children by an independent safety monitoring committee is also an important issue to consider [6,7]. PDCO is legally required to publish its opinions and decisions on PIPs, waivers and deferrals (commercially confidential information can be deleted). ERA-NET PRIOMEDCHILD suggests that the PDCO's decision process on waivers would gain to be more transparent. Besides PDCO's required studies may have to be reconsidered in terms of ethical and economical feasibility. The involvement of all stakeholders earlier in the process may be an opportunity.

The United-States (US) approach for paediatric authorization seems pragmatic and more flexible than in Europe. Food and Drug Administration (FDA) asks to pharmaceutical companies a complete Pediatric Development Plan (equivalent to PIP in EU) providing any sufficient safety data base from adult population. When an off-label drug is used for a long period, US authorities give a paediatric authorization based on i) the number of paediatric patients already treated, ii) available efficacy and safety data collected among a rather large paediatric population, iii) the life duration of the off-label product use, iv) adequate safety data base in adults. Specific and justified studies are demanded only if those points are not met. Interactions between FDA and EMA should benefit from each other's experience the priorities for paediatric drug research being obviously set by the need of the patients, not by market considerations [4,8-12].

### Increasing responsibilities for researchers

Beneficence and responsibility go along, they are two major and significant ethical issues to be found in the new Regulation [2] (having better medicines for children is a *Beneficence*) and in the Directive-2001/20/EC [13] (having a reinforced protection for children involved in clinical trials implies a higher *Responsibility *for the researcher). Article 4 on "Clinical trials on minors" gives specific rules to reinforce the protection of minors participating to clinical research. Most of these stipulations are related to the informed consent process. Specific protection of minors implies strengthened protection and possibly strengthened legal sanctions. Some countries sanction researchers who fail to obtain the informed consent (see article L. 1126-1 of the French Law "Code de la Santé Publique" [14] providing a three-year jail term for infringement of the consent procedure). From a legal point of view, the patient's consent (and/or that of his/her legal representative for minors) to participate is mandatory. Nevertheless the consent form does not prevent the researcher from civil prosecution if a child is subjected to damage during a clinical research. For vulnerable people, an objective civil liability is recognized by many European legal courts [15].

### From informed consent to shared consent

The informed consent should be obtained by respecting the autonomy principle, i.e., giving complete and intelligible information to the patient (and/or his/her legal representative for a minor) allowing him/her to take a free decision. Many parents openly admit that the informed consent process is useful, albeit often confusing. They find discussions more helpful than the consent documents [16]. The decision for trial participation is influenced by parental, child, trial and physician factors [17]. Families can also - and often do - ask their general practitioner, relatives or various people for advice [18]. While collecting the written consent in the case of vulnerable people, the Whereas 4 of the Directive-2001/20/EC [13] draws attention to the treating doctor's cooperation "the written consent of the patient's legal representative, given in cooperation with the treating doctor, is necessary before participation in any such clinical." So far, the role of the treating doctor seems underestimated or ignored and more attention should be paid to define his/her specific role to reinforce the protection of vulnerable subjects. Researchers should build better relationships with paediatricians/family doctors [18,19]. Even a tricky concept to apply in practice, concomitant oral information by an independent trustworthy physician chosen by the family may be an opportunity for substantially improving parental understanding of the particular benefits, risks and alternatives of a trial. Providing enough time to come to the decision of consent [20,21] is also part of the precautionary principle [22].

### A pragmatic approach for assent

Despite the mandatory direct participation of children in a shared-consent procedure, in most cases consent is obtained after discussions with and agreement of parents only while the children view point is often not taken into account [23]. The neonate population represents the most vulnerable of all paediatric age groups and requires even more careful review [24]. The definition of the age of consent is a controversial topic. Important discrepancies on the consent/assent process exist among EU-member-states [25,26]. Wendler [27] recommends that until instruments are developed to assess the assent capacity of individual children (ability to understand the research in question), 14 should be the threshold age for assent. The Ethics Working Group of the Confederation of European Specialists in Paediatrics [28] considers that the ability to understand the aim, possible benefits and risks of a research study can be expected at the earliest from the age of 9 onward. Yet, one must remember that the age for criminal liabilities is 7 in Switzerland, 11 in the United Kingdom and 14 in Italy. In some extent it sounds such as a paradox that minors of the same ages in one country can be recognized as criminals but could not have their assent taken into account for clinical trial. To conclude on this controversial issue, we strongly recommend referring to the Oviedo Convention Additional Protocol stipulation, i.e., "*specific rules aimed at assuring that minors' opinions should increasingly carry more weight in the final decision*" [29].

### From indirect to direct benefit

A clinical trial on minors may be undertaken only if benefit for this group of patients is obtained [13]. However, it remains still open the question whether or not it is acceptable to expose children to some research risks for the benefit of others. The results of the survey conducted by Wendler and Jenkins [30] support the acceptability of such an exposure. According to Westra et al [31] the absolute limit of minimal risks and minimal burdens stipulated by the EU recommendations may prohibit important appropriate research. The involvement of children in research that will not directly benefit from them clearly poses an ethical dilemma. To get a fair informed assent/consent, the minor and his/her representative should have also had a clear idea of the possible direct effects [32] in accordance with the following ethical principle that "patients' interest should always prevail over science/society" [33]. The clinical physician should consider the patients and the trial NOT in a holistic way but on a careful case-by-case assessment.

### Risk threshold and legal risk

The application of the rules of good clinical practice when performing clinical trials of drugs for human includes the requirements for insurance policies safeguarding participants [2,13]. A more comprehensive approach to define the risk among stakeholders is necessary. It could be defined according to the type of research (medicinal products requiring a marketing authorization, post-marketing studies, interventional or non-interventional clinical trials) or to the type of trials (invasive, non-invasive, Phase I to IV). Clinical trial monitoring according to a risk-based approach would be more appropriate for off-patent products. It would entail a substantial reduction of workload and cost, particularly for academic institutions that run low-risk studies using marketed drugs [5,34]. To define the legal risk of a clinical trial, insurance companies have a more quantitative and simplified approach of the risk/benefit assessment than scientists. The risk increases with the number of patients involved in a trial and with the scope of the coverage (e.g., as with innovative products). Usually, contracts cover for a certain number of clinical trials conducted by the same sponsor during a defined period of time. Insurance companies are trying to reduce the complexity of the risk assessment by selecting a set of specific risk factors. As the data communicated by sponsors are usually restricted for confidentiality reasons, this task is not easy. Estimating the delay of the occurrence of a prejudice (damage that may occur later on [35]) is another issue.

### Ethical use of placebo

The placebo use is going to be accepted at regulatory agency level in view of limiting the number of experimental subject. Indeed, regulatory and ethical guidelines highlight that in some instances the judicious use of placebo remains essential to demonstrate the efficiency of new medicines (Table 1) [26,36,37]. Nevertheless, the ethics of placebo-controlled paediatric studies causes concern [10]. One of the reasons is that most paediatricians/family doctors have no knowledge of drug trials (methodological and ethical aspects). There is also a poor awareness and understanding of paediatrics randomized clinical trials by parents [16,38]. Placebo is however the standard control applied for efficient study design such as added on and withdrawal design [10,37].

**Table 1:** Ethically acceptable use of Placebo

References	Ethics of placebo-controlled trials
[36]	When there is no commonly accepted therapy for the condition and the new medical entity is the first one that may modify the course of the disease.

[10]	When patients have failed to respond to standard treatment, they might be randomised to a new treatment or a placebo because they have exhausted alternative options

[36]	When the commonly used therapy is of questionable efficacy.

[36]	When the commonly used therapy carries a high frequency of undesirable side effects and the risks may be significantly greater than the benefits.

[36]	When the placebo is used to identify incidence and severity of undesirable side effects produced by adding a new treatment to an established regimen.

[36]	When the disease process is characterized by *frequent, spontaneous exacerbations and remissions *and the efficacy of the therapy has not been demonstrated

### Ethics committee with paediatric expertise

A clinical trial on minors may be undertaken only if the ethics committee has endorsed the protocol [26]. The ethics committee which is an independent body including health professionals must assess the relevance and advantages of the clinical trial, the risk/benefit ratio, the quality of the healthcare institution and whether the research subjects (or their legal representatives) have been properly informed. According to the recommendations of the ad hoc group, the ethics committee should include paediatric experts such as physicians with paediatric qualification, paediatric ethicists, and paediatric pharmacologists. In addition the experts should demonstrate at least some years of experience in paediatric care and direct experience of clinical trials with children in similar age groups [26]. Recruitment or co-option of members with adequate experience seems non obvious for some ethics committees as qualified researchers in sufficient number are not available among EU-member-states [25,39-41]. Newly emerging areas such as paediatric research might face problematic workloads, responsibilities and costs.

### Heterogeneity of ethics committee procedures among EU-member-states

In case of multicentre clinical trials carried out in more than one member state simultaneously, an ethics committee's single opinion must be given for each EU-member-state implicated with the multicentre trial [13]. Ethics committees are heterogeneous thus offering multiple views and judgments. Should be assessed local features such as the quality of the investigation site, appropriateness of the information document as formulated in national language, issues connected to national legislation (data protection, liability). Therefore, trials could be delayed if they are to receive each local ethical approval. Actually, clinical researchers get the impression of unnecessary duplication of efforts with multiple ethics committees for the same trial. However, centralization or standardization may rationalize the application process entailing that the final decision might be taken without an in-depth consultation of people who are directly involved in or aware of local ethical issues. And regular communication with local ethics committees is essential to guaranty the independence of the final decision and as a consequence the protection of vulnerable subject. Yet these multiple consultations should not create barriers or unnecessary delays [20,42,43]. An opportunity could be to nominate an ethics committee coordinator/facilitator who should guaranty the quality of the discussions between national ethics committees having in mind the need for speed to get the final approval. This ethics committee coordinator/facilitator should not be an additional administrative step in the evaluation process and should be ethically and economically independent from the sponsors of the study (may be a representative of one of the involved ethics committees).

### ERA-NET Priomedchild final recommendations

The ERA-NET PRIOMEDCHILD final recommendations to facilitate the development of clinical research with children are summarized in Table 2.

**Table 2:** ERA-NET PRIOMEDCHILD recommendations for framework conditions facilitating medicines for children research

Identified Issues	ERA-NET PRIOMEDCHILD recommendations
**Decision-making process**	Open the governance to a collective involvement of all stakeholders including a more active involvement of parents' associations and viewpoints of children as soon as the protocol elaboration begins.

**Ethics Committees' expertise**	Develop criteria for accreditation of paediatric ethics committees in EU countries and identify in EU-member-states the qualified ethics committees to review paediatric research.

**Education & Training in clinical pharmacology**	Promote education and training in paediatric research with formal EU academic recognition. Train a team of experts in paediatric research independently remunerated for the review of study protocols. Implement dedicated units with qualified and trained people in paediatric clinical research within public hospitals, scientific societies, regulatory authorities and ethics committees.

**Multinational regulatory approvals**	Create the function of independent ethics coordinator to facilitate communication between local/national local ethics committee for multicentre/international clinical trials.

**Time to come to the decision of consent**	Establish guidelines i) to allow sufficient time to consider pros and cons for child participation into a clinical trial including the possibility to ask for external advice ii) to give the opportunity to parents and children to debate on ethical issues in dedicated places with trusty persons before and during child's involvement in clinical trials.

**Integrated safety data- base**	Systematic collection, monitoring and analysis of clinical trials data with medicines provided by sponsors and periodic publishing of the reports being accessible to professionals and to the general public.

**Sharing strategies and plans**	Harmonize the interaction between EMA and FDA in order to agree on paediatric development programmes or waivers to avoid exposing children to unnecessary trials.

**Adapted methodologies & design**	Implement urgently methodologies and strategies using the lowest possible number of subjects as outlined in the EMA guidelines on clinical trials in small populations.

**Unmet need for children**	Reduce the prevalence of unmet need for children research by i) amending the regulation to compel the development of new indications and formulations for off patent products, ii) supporting adequate public-private partnership for off label drugs research, iii) developing specific procedures or guidelines adapted to non-profit research.

**IPR issues**	Establish a EU legal framework for the Intellectual Property Right (IPR) issues arising from private-public partnership in paediatric clinical research

## Conclusions

In order to move from "research as a potential damage" to "research as health opportunity for children" European paediatric research should change in a multidisciplinary approach, taking advantage of the 30-year experience US approach. A better communication/sensitization of parents concerning the necessity of clinical research with a fair distribution of responsibilities among the stakeholders will increase parents' willingness to participate in trials. Sufficient time to consider pros and cons for child participation into a clinical trial should be given to the family including the suggestion to ask for external advice. To avoid supplementary delays due to the necessity to submit the study to several ethics committees the nomination of an independent ethics coordinator should be an opportunity. Extra funds, human resources, adequate research structures, education and training are necessary to optimise the process of implementing clinical trials with children.

## Competing interests

The authors declare that they have no competing interests.

## Authors' contributions

ALK and PP managed and coordinated the WP3 and wrote the scientific reports for the deliverables of ERA-NET PRIOMEDCHILD Work Package 3 (WP3). SB analysed and evaluated legal and ethical aspects of the regulation. ODCA analysed and evaluated regulatory aspects of the regulation. EB contributed the discussion of ethical and legal aspects of WP3 results. IL contributed the discussion of regulatory aspects of WP3 results. DA coordinated the overall ERA-NET PRIOMEDCHILD project and actively participated to all WP3 meetings. All authors contributed to the intellectual content and approved the final version.
